# HOPON (Hyperbaric Oxygen for the Prevention of Osteoradionecrosis): a randomised controlled trial of hyperbaric oxygen to prevent osteoradionecrosis of the irradiated mandible: study protocol for a randomised controlled trial

**DOI:** 10.1186/s13063-017-2376-7

**Published:** 2018-01-10

**Authors:** Richard Shaw, Christopher Butterworth, Binyam Tesfaye, Matthew Bickerstaff, Susanna Dodd, Gary Smerdon, Seema Chauhan, Peter Brennan, Keith Webster, James McCaul, Peter Nixon, Anastasios Kanatas, Paul Silcocks

**Affiliations:** 1 CRUK Liverpool Cancer Trials Unit, Department of Molecular and Clinical Cancer Medicine, University of Liverpool and Aintree University Hospital NHS Foundation Trust, Liverpool, UK; 2 CRUK Liverpool Cancer Trials Unit, Department of Molecular and Clinical Cancer Medicine, University of Liverpool, Liverpool, UK; 3 Department of Biostatistics, University of Liverpool, Liverpool, UK; 4 Devon Diseases Research Centre, Plymouth, UK; 5 Portsmouth Hospitals NHS Trust, Portsmouth, UK; 6 Queen Elizabeth Hospital, University Hospitals Birmingham NHS Foundation Trust, Birmingham, UK; 7 Queen Elizabeth University Hospital, Glasgow, UK; 8 Leeds Dental Institute, Leeds, UK

## Abstract

**Background:**

Osteoradionecrosis of the mandible is the most common serious complication of radiotherapy for head and neck malignancy. For decades, hyperbaric oxygen has been employed in efforts to prevent those cases of osteoradionecrosis that are precipitated by dental extractions or implant placement. The evidence for using hyperbaric oxygen remains poor and current clinical practice varies greatly. We describe a protocol for a clinical trial to assess the benefit of hyperbaric oxygen in the prevention of osteoradionecrosis during surgery on the irradiated mandible.

**Methods/design:**

The HOPON trial is a phase III, randomised controlled, multi-centre trial. It employs an unblinded trial design, but the assessment of the primary endpoint, i.e. the diagnosis of osteoradionecrosis, is assessed on anonymised clinical photographs and radiographs by a blinded expert panel. Eligibility is through the need for a high-risk dental procedure in the mandible where at least 50-Gy radiotherapy has been received. Patients are randomised 1:1 to hyperbaric oxygen arm (Marx protocol) : control arm, but both groups receive antibiotics and chlorhexidine mouthwash. The primary endpoint is the presence of osteoradionecrosis at 6 months following surgery, but secondary endpoints include other time points, acute symptoms and pain, quality of life, and where implants are placed, their successful retention.

**Discussion:**

The protocol presented has evolved through feasibility stages and through analysis of interim data. The classification of osteoradionecrosis has undergone technical refinement to ensure that robust definitions are employed. The HOPON trial is the only multi-centre RCT conducted in this clinical setting despite decades of use of hyperbaric oxygen for the prevention of osteoradionecrosis.

**Trial registration:**

European Clinical Trials Database, ID: EudraCT200700622527. First registered on 5 November 2007.

**Electronic supplementary material:**

The online version of this article (doi:10.1186/s13063-017-2376-7) contains supplementary material, which is available to authorized users.

## Background

Osteoradionecrosis (ORN) describes the process where irradiated bone undergoes necrosis and becomes exposed through the investing soft tissues for a period of at least 3 months [1–3]. An important precipitating factor for mandibular ORN is surgical trauma, commonly dental extractions or implant placement following head and neck radiotherapy, but ORN can also occur spontaneously. ORN is painful and debilitating, often requiring surgical resection of the jaw and complex multidisciplinary management [4]. The morbidity and mortality of ORN is significant and treatment outcomes often unsatisfactory. Post-radiation extractions should, self-evidently, be performed atraumatically and antibiotics are commonly prescribed [5], but there is a paucity of high-quality evidence to guide best practice in the prevention of ORN.

Preventive approaches include pre-radiotherapy extraction of teeth and the use of hyperbaric oxygen (HBO) treatments or prophylactic antibiotics for post-radiotherapy extractions [6]. The overall incidence of ORN among post-radiotherapy patients is not certain but may have declined with improvement of radiotherapy techniques including intensity-modulated radiotherapy (IMRT) over recent decades [7]. However, the incidence of head and neck cancer, the proportion of cases receiving radiotherapy, and prognosis are all increasing [8], contributing to an expansion in the ‘at risk’ population where ORN prevention must be addressed. The risk of ORN with dental extractions is higher in the posterior mandible, with radiotherapy doses higher than 60 Gy (or with brachytherapy) and in smokers [9].

There have only been limited trials of prophylactic HBO. The single-centre randomised controlled trial (RCT) by Marx et al. [10] showed a significantly lower incidence of ORN after post-radiotherapy dental extractions in the HBO group when compared with the control group. There were two cases of ORN in 37 patients (5.4%) receiving HBO undergoing 156 extractions, compared with 11 cases in 37 patients (29.9%) undergoing 135 extractions receiving prophylactic penicillin, resulting in a number needed to treat (NNT) of 4. In a non-randomised retrospective study, Vudiniabola et al. [11] showed that of 29 patients who received pre-extraction HBO, 1 (3.4%) developed ORN; and of 7 patients who did not receive HBO, 1 (14.3%) developed ORN. Despite improvements in radiotherapy since the time of these studies, and evidence of a decreasing incidence of ORN [12], prophylactic HBO to prevent ORN has remained a standard of care for high-risk dental extractions [13].

HBO is not available to all patients as it requires proximity to a chamber, it is costly (approximately £4000–6000 per course) and sometime logistically problematic, requiring multiple daily visits. A typical protocol for prevention dictates 45 h of treatment over 30 compressions [10]. There are also risks associated with HBO therapy. A comprehensive recent safety review [14] noted common temporary visual problems, Eustachian tube problems 2%, claustrophobia 2% and seizure < 0.01.

A survey of UK practice revealed that, in a high-risk extraction of a lower mandibular molar, 33% ‘never’, 41% ‘sometimes’ and 26% ‘usually’ or ‘always’ prescribe prophylactic HBO [15]. Questionnaires of attitude to RCTs for HBO revealed that 93% of responders would wish to recruit such patients into multi-centre RCT [16]. Additionally, there is a lack of agreement on HBO protocol, with some UK patients referred for treatment in centres using non-standard pressures [17], lower than the typical 2.4 atmospheres (ATA) recommended in most ORN publications. Current National Institute for Health and Care Excellence (NICE) guidelines on the management of ORN recommend the use of HBO only as part of a clinical trial [18].

An additional question regarding the efficacy of HBO surrounds the placement of osseointegrated implants into the irradiated mandible, with the aim of preventing ORN or optimising bony healing and implant retention. Survey data again suggest wide disparity in practice [15] in the UK. No RCT has been carried out in this setting, and there are conflicting data from retrospective case control series [19, 20] as to whether HBO may enhance survival of implants in irradiated bone.

## Objective

The objective of the HOPON trial is to determine the benefit of HBO in the prevention of ORN subsequent to a surgical procedure in the ‘at risk’ irradiated mandible. The procedures included are high-risk dental extractions and the placement of implants. Additional outcomes are to document the incidence of ORN in the control group, the outcomes where ORN is diagnosed, and the retention of implants. The trial will also measure the effect of HBO in post-operative pain and effects on quality of life. The HOPON phase III trial protocol described here has evolved from the feasibility objectives in a preceding HOPON feasibility study.

## Methods/Design

### Design

HOPON is a prospective, multi-centre, randomised controlled, phase III trial to assess the effectiveness of preventing ORN after surgical procedures in the irradiated mandible. Although the patients and investigators are unblinded, the primary endpoint is assessed by a blinded panel for the presence and grade of ORN. Other endpoints are unblinded as they are recorded by either the patients or site investigators.

### Study population and eligibility

Patients who meet the eligibility criteria (Table 1) will be recruited either from routine review following the treatment of head and neck cancer, or following referral for oral rehabilitation. Detailed information on the benefits and risks of the study will be provided to the patients, including the provision of HBO at the nearest suitable trial chamber.

**Table 1:** Eligibility criteria

Inclusion criteria	Exclusion criteria
Age > 18 years Prior radiotherapy to mandible > 50 Gy No evidence of cancer recurrence Condition requiring surgery to mandible: Extraction of premolar or molar Implant placement Provision of written informed consent in competent patient	Prior diagnosis of ORN of the mandible Prior HBO therapy for any indication Any previous prescription of systemic bisphosphonates, pentoxyphylline or tocopherol Pregnancy Contraindications to HBO: Lung disease (severe COPD or bullae) Middle ear Claustrophobia

*COPD* chronic obstructive pulmonary disease, *HBO* hyperbaric oxygen, *ORN* osteoradionecrosis

### Randomisation and blinding

**Fig. 1:**
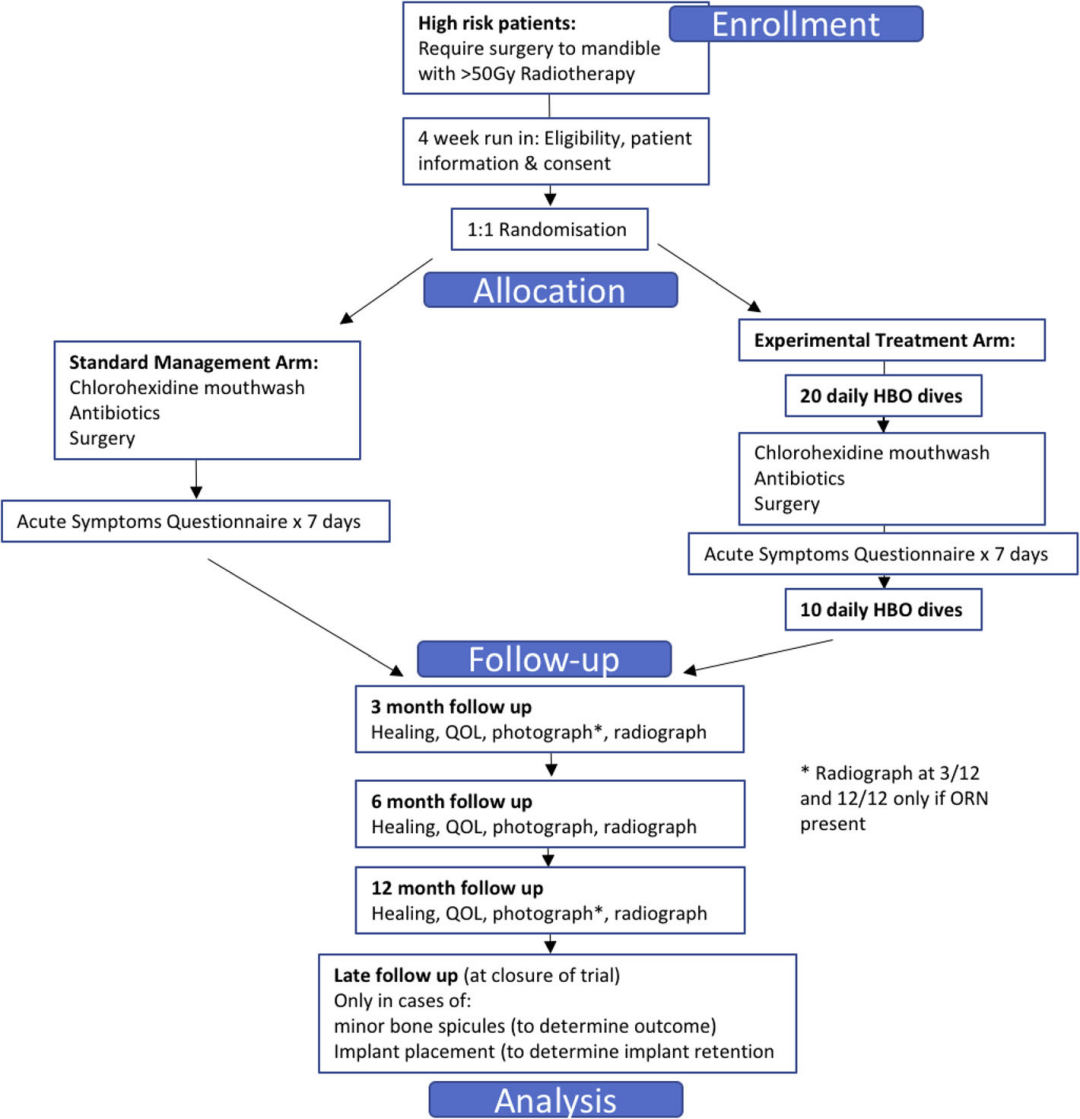
Hyperbaric Oxygen for the Prevention of Osteoradionecrosis (HOPON) trial schema (Consolidated Standards of Reporting Trials (CONSORT) format)

Patients who meet eligibility criteria and have given informed consent will be randomly assigned by the Cancer Research UK Liverpool Cancer Trials Unit (LCTU). Randomisation will be in a 1:1 ration between HBO arm and control (non-HBO) arm, stratified by recruiting centre. The randomisation code list will be generated by the LCTU trial statistician by means of block randomisation [21] with randomly varying block length. The clinical team will be informed of the allocation of each patient by fax. Allocation of treatment is unblinded to local investigators and patients. The trial schema is shown in Fig. 1.

### Trial intervention and control treatments

The supplier of the oxygen to be used will be dependent on standard practice within each hyperbaric unit. Treatment will be administered based around a version of the Royal Navy Therapeutic Table 66 – Repeat Hyperbaric Oxygen Therapy [22]. The patient undergoes pressurisation to 2.4 ATA at a tolerable rate. The patient undergoes decompression after 100% oxygen has been breathed at 2.4 ATA for a total of between 80 and 90 min. Air breaks while at 2.4 ATA may be introduced routinely or as required. The decompression is scheduled to control satisfactorily any risk to the patient and, if present, to the in-chamber attendant. Patients should breathe oxygen at an inspired partial pressure greater than 2.0 ATA for no more than 110 min during each individual treatment.

Patients in both arms of the trial are given chlorhexidine mouthwash and antibiotics. Pre- and post-operative chlorhexidine mouthwash 0.2% is used in a volume of 10 ml (i.e. one capful) washed around the mouth for around 1 min and spat out, three times daily (tds) for 5 days post-operatively. In case of chlorhexidine allergy, warm salt mouthwash at 1 teaspoon per cup of warm water is used. Orally administered antibiotics comprise amoxicillin 3 g 1 h pre-operatively (or 1 g administered intravenously) and 250 mg tds for 5 days post-operatively. In case of penicillin allergy this will be: 600 mg orally administered clindamycin (either 600-mg tablets orally (or the same dose of a 75-mg/5 ml suspension if tablets not tolerated) given 1 h pre-operatively (or 600 mg administered intravenously at the time of surgery) and 200 mg metronidazole orally tds for 5 days post-operatively.

### Outcome measures

The primary outcome measure is the diagnosis of ORN 6 months following surgery as determined by a blinded central review of clinical photographs and radiographs as well as the site investigator’s assessment. This blinded review is carried out by an expert panel, with access to paired clinical photographs and radiographs (orthopantomogram). Additionally, where possible, any area of exposed bone is presented in context of an in-field rule and/or site investigator’s measurement on the case report forms. The diagnosis of ORN at 3 and 12 months are secondary outcome measures, but determined in a similar way, the only difference being that radiographs will only be taken at these time points if ORN has been clinically diagnosed. A summary of the outcome measures and their time points is presented in Table 2.

**Table 2:** Outcome measures, time of assessment and method of assessment

Outcome measure	Timing	Method of assessment
Primary outcome		
Presence of ORN	6 months	Blinded review of radiograph, clinical photograph with corroboration from site investigator’s assessment in CRF (and dimensions of any exposed bone)
Secondary outcomes		
Presence of ORN	3 and 12 months	As primary endpoint, but without radiographs unless ORN clinically evident
Severity of ORN	3, 6 and 12 months	Blinded review of radiograph, clinical photograph, site investigator’s assessment (and dimensions of any exposed bone) using modified Notani Score [23, 24]
Quality of life	3, 6 and 12 months	University of Washington head and neck cancer questionnaire
Pain following surgery	3, 6 and 12 months	Patient-reported on a Visual Analogue Scale and use of analgesia
Acute symptoms	Days 1 thru 7 post surgery	Patient-reported Likert scale for pain, swelling, trismus, normalcy of diet
Assessment of implants (where relevant)	At closure of trial	Casenote review, by loss of any implant placed as part of trial
Late follow-up of MBS cases (where relevant)	At closure of trial	Casenote review, by severity of ORN using modified Notani Score [23, 24].
Safety of HBO related to cancer recurrence	Within 12 months of trial treatments	SAE reporting.
Safety of HBO, otherwise	Within 28 days of HBO	AE/ SAE reporting of symptoms related to hyperbaric treatment

*AE* adverse event, *CRF* Case Record Form, *HBO* hyperbaric oxygen, *MBS* minor bone spicules, *ORN* osteoradionecrosis, *SAE* serious adverse event

**Fig. 2:**
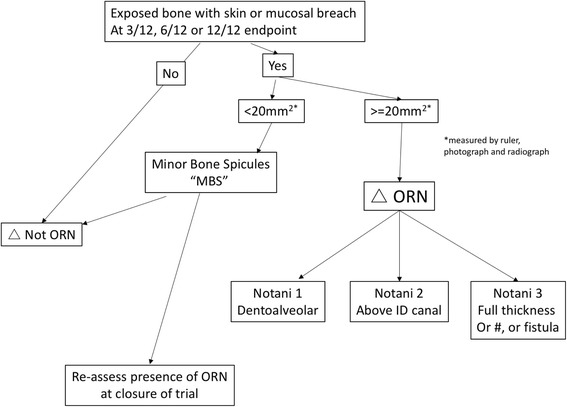
Criteria for diagnosis of osteoradionecrosis (ORN) in the Hyperbaric Oxygen for the Prevention of Osteoradionecrosis (HOPON) trial [24]. ORN, further classified [23]: Notani 1: ORN confined to alveolar bone. Notani 2: limited to the alveolar bone and/or above the level of the inferior alveolar canal. Notani 3: ORN under the lower part of the inferior alveolar canal, with fistula or bone fracture

The definition and classification of ORN used in the trial, based on that of Notani [23], has been subject to a technical refinement through protocol amendment following blinded analysis of the HOPON feasibility data [24], and is presented in Fig. 2. This was required to resolve inconsistencies which arose in cases where very small areas of exposed bone were seen: ‘minor bone spicules’ (MBS) were apparent, which occurred in 19% of patients in this initial analysis. In reporting cases with MBS, some trial investigators took a pragmatic position in judging MBS as clinically unimportant, likely gradually healing, and not reflecting progressive ORN. Others classified as Notani 1 ORN based on rigid definitions in common clinical use. When MBS was added as an additional distinct category to the classification (as < 20 mm^2^) this ambiguity was resolved and agreement between observers was achieved. This refinement was adopted by the protocol after discussion with trial oversight committees and the relevant regulatory bodies. A working assumption was made, after consultation, to group MBS with the healed (‘not ORN’) cases, but another outcome measure of the trial will be to test this assumption against the outcomes for later time points. For the purpose of the HOPON trial, radiological signs alone, or pain alone, are not considered ORN unless accompanied by a breach in overlying skin or mucosa.

As the primary endpoint of the trial is dependent on the availability and quality of radiographs and clinical photographs, a quality-control protocol is adopted. All new centres recruiting patients will have the first sets of images audited, and then 10% thereafter. These will be scored as adequate/inadequate to assess the presence of ORN by an appropriate panel assembled, viewed independently by three qualified clinicians, on reviewing anonymised images. Any centres submitting photographs of inadequate standard will be offered advice and training.

**Fig. 3:**
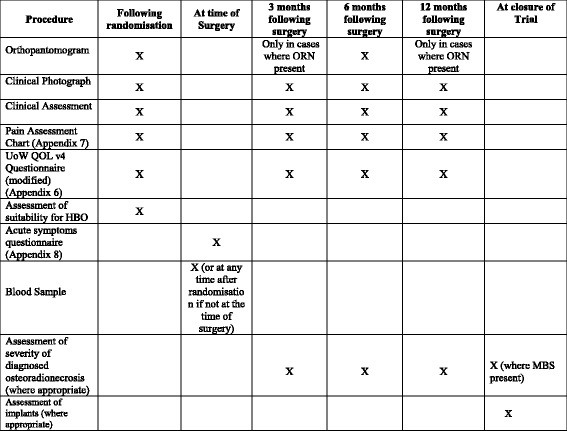
Standard Protocol Items: Recommendations for Interventional Trials (SPIRIT) trial schedule

The schedule of enrolments, interventions and assessments is summarised using the Standard Protocol Items: Recommendations for Interventional Trials (SPIRIT) schedule as Fig. 3; and the SPIRIT Checklist provided as Additional file 1.

### Safety and adverse event reporting

Standard definitions and procedures for the management of adverse events (AE), serious adverse events (SAE), serious adverse reactions (SAR) and suspected unexpected serious adverse reactions (SUSAR) are adopted within the trial. AEs and reactions are reported using relevant Case Record Form (CRF), whether related to treatment or disease, within 28 days following the last trial treatment. This will be antibiotics and mouthwash in the control arm, and HBO in the treatment arm. In addition, a new diagnosis of cancer or recurrence of cancer will be reported as an SAE for the duration of the patient’s involvement in the study up to 12 months after surgery, irrespective of whether this is related to head and neck cancer, or the original indication for radiotherapy.

## Statistical considerations

### Sample size

Assuming an ORN rate of 5% in the HBO arm at 6 months following surgery consistent with an earlier RCT [10], 103 evaluable patients per group would provide 80% power to detect an odds ratio of 0.23 (equivalent to detecting an absolute difference of 13.5% between treatment arms) at a 5% two-sided significance level. An estimated dropout rate was extrapolated from feasibility data at 7%, resulting in an estimate of 221 patients to be recruited in total.

### Analysis plan

It is not planned to fully adhere to the intention-to-treat (ITT) principle of ‘analysing as randomised’ because patients do not have the intervention (HBO or standard care) until they have been randomised and had surgery arranged. Feasibility data revealed a somewhat higher dropout rate after randomisation in the HBO arm because of the increased opportunity available in the time taken to schedule HBO. Furthermore, the clinical question to be answered is the effect of HBO, but only among those receiving surgery, and not the effect of HBO in itself. Demographic and clinical factors for the HBO and control groups will be presented both ‘as randomised’ and also ‘as receiving surgery’. A multiple logistic regression analysis will be performed to confirm that these factors remain jointly uninformative for receiving surgery.

The primary test of efficacy in terms of risk of ORN at 6 months will be carried out using an exact logistic regression including a fixed term for treatment arm. The null hypothesis is that inclusion of HBO treatment pre-surgery is not more effective than standard care alone; that is, the odds ratio is not statistically different from 1, while the alternate hypothesis is that HBO treatment is superior to standard care with an odds ratio of 0.23 or less. The test will be two-sided and a *P* value of less than 0.05 will be declared statistically significant. Two-sided 95% confidence limits for the odds ratio will be presented for consistency with the significance test. Significance tests for secondary endpoints will also be two-sided at 5% accompanied by 95% two-sided confidence intervals.

### Interim analyses

A feasibility stage was incorporated into the original funding application with endpoints of ability to recruit, randomise and complete trial protocols for 50 patients within 2 years of commencement. Following this, regular analyses of trial data by the Independent Safety Data Monitoring Committee (ISDMC) review occur at 12-monthly intervals to assess recruitment rates and toxicity. A single, formal interim analysis will be carried out when 100 dental extraction patients have been followed up to the primary endpoint at 6 months. The Peto stopping rule will be implemented for the primary efficacy outcome (ORN rate at 6 months following surgery), and a futility analysis will also be carried out.

### Sensitivity analyses

These will consist of:

1. Assessing the effects of assuming that:
  - all pre-surgery dropouts would have developed ORN
  - no pre-surgery dropouts would have developed ORN
  - all pre-surgery dropouts would have developed ORN with risk for their arm
  - all pre-surgery dropouts would have developed ORN with mean risk across both arms
2. Assessing the effect of adjustment in analysis of the primary outcome for radiotherapy dose, age, sex, tobacco and alcohol use
3. Inclusion of multiply imputed primary endpoint responses for all randomised patients
4. Comparison of results using the site principal investigator’s (PI’s) original assessment of ORN (as with the primary analysis, this will include assessment of centre effects)
5. Analysis of ‘blinded review’ ORN after including MBS vs excluding cases with only MBS

### Translational research

In addition to the routine trial assessments, translational blood samples will be taken either at the time of surgery, or at any time after randomisation. Sample collection kits will be sent to all participating sites and the samples collected and stored to Good Clinical Laboratory Practice (GCLP) standard at the University of Liverpool. These samples will be used at a future date to discover/evaluate potential biomarkers of the genomic determinants of ORN [25].

## Trial oversight and regulatory arrangements

### Trial Management Group (TMG)

This comprises the chief investigator (CI), other lead investigators (clinical and non-clinical) and members of the LCTU. The TMG will be responsible for the day-to-day running and management of the trial and will meet approximately monthly.

### Trial Steering Committee (TSC)

The TSC will consist of the TMG plus three independent members, two patient representatives and an independent chair. The TSC will provide overall supervision for the trial and provide advice through its independent chair. The ultimate decision for the continuation of the trial lies with the TSC.

### Independent Safety Data Monitoring Committee (ISDMC)

The ISDCM will be responsible for reviewing and assessing recruitment, interim monitoring of safety and effectiveness, trial conduct and external data. The ISDMC will first convene prior to trial opening and will then define frequency of subsequent meetings (at least annually). The ISDMC will provide a recommendation to the TSC concerning the continuation of the study.

### Sponsorship

The HOPON trial is co-sponsored by the University of Liverpool and Aintree University Hospitals NHS Foundation Trust.

### Registration

The HOPON trial is registered with the European Clinical Trials Database (EudraCT 2007-006225-27).

### Clinical trial authorisation

The HOPON trial uses oxygen which is classified by the Medicines and Healthcare Products Regulatory Authority (MHRA) as an Investigational Medicinal Product. The trial has received a clinical trial authorisation by the MHRA under the Medicines For Human Use (Clinical Trials) Regulations 2004 S.I. 2004/1031 (MHRA **r**eference: 04196/0010/001-0019).

## Discussion

Several areas of controversy have arisen during the development of the HOPON protocol. The first of these is the need for blinding. Some comparable trials in other fields of late radiation toxicity have used a sham HBO arm with the aim of producing a double-blind trial [26–28]. The sham HBO arm used in such trials varies considerably. One approach [26] utilises a breathing mixture of 9% oxygen and 91% nitrogen so that at 2.4 ATA, arterial PaO_2_ is similar to breathing 21% oxygen at ambient air pressure. This, however, still exposes patients to the documented risks of hyperbaric pressure, and is by some considered unethical because of the implied issues of safety without any chance of benefit. Another approach used 40% oxygen and 60% nitrogen [28] providing an alternative lower ‘dose’ of oxygen treatment and correlation of outcome rather than a true control arm. More recently [27, 29] sham HBO arm using air at 1.1 ATA [29] or 1.3 ATA [27] have been used to provide a minimal increase in partial pressure of oxygen (PpO_2_) while providing the sensations of compression. Regardless of mode of delivery, the provision of sham HBO treatments are equivalent in cost to those delivered on the treatment arm which, given finite resources, may impact the cost-effectiveness of such research. Finally, it is also uncertain whether the HBO chamber staff could ever be effectively blinded as they remain in control of the chamber. It is, therefore, possible that patients become unblinded when treated for over 30 treatment sessions, particularly as these sham treatments are delivered at a regular and different daily session to the oxygen treatments.

The need for a blinded trial using sham treatments is considered desirable in trials where the primary endpoint is subjective or employ patient-reported outcome measures. In the HOPON trial the primary outcome is the presence of ORN, which is considered more objective, and additionally can be assessed on clinical photographs and radiographs at a remote time and location, by a panel of investigators who *are* blinded to the treatment arm received. However, the unblinded trial design for HOPON does introduce the possibility of reporting bias in some of the more subjective secondary outcomes such as quality of life or pain. Further, the unblinded design of the trial and need for extra treatments in the HBO arm introduces an inequality in time between randomisation and surgery for that arm. This potential methodological concern means that the time points commence at the time of surgery, rather than the date of randomisation, where it might be argued that only at randomisation are the two groups truly comparable.

Another difficulty that has arisen during the feasibility stage of the trial is the difficulty in robust allocation of some early stages of ORN. These have subsequently been classified as minor bone spicules (MBS), but are not reflected within any of the classifications of ORN currently in use. This difficulty is presumably not unique to the HOPON trial, yet it is difficult to ascertain from other similar studies how such cases have been dealt with. This is a problem created by taking a working ‘textbook definition’ of a clinical condition and extrapolating it as a primary outcome measure in a clinical trial. Because of the frequent (19%) finding of MBS during a blinded interim data review, it was logical to introduce an additional class to reflect this, the justification for which has been separately published [24]. To establish if any of the MBS cases in the HOPON trial will progress to clinically significant ORN, there is a secondary outcome measure of outcome of such patients at the conclusion of the trial. As a means to avoid any ambiguity, therefore, the use of the Notani classification with the additional category of MBS has been employed in order to avoid subjectivity and to enhance reliability and consistency of reporting.

Further concerns were around how to deal with missing data, dropouts and the effect of differing complexity and length of treatment between the two arms. While ITT analysis is usual practice for the primary analysis in superiority trials, in this trial ‘analysing as randomised’ would not answer the clinical question which relates to a fixed time following surgery, in other words the intended primary analysis is essentially on a per-protocol or treatment-received basis. While unusual, this is one of the circumstances in which, as Piantadosi [30] has pointed out, too strict adherence to the ITT principle is unhelpful or inappropriate.

The HOPON trial design has, therefore, evolved through analysis of feasibility data and through blinded interim data analysis. This is the only formally conducted, multi-centre, RCT conducted in this field that the authors are aware of, and it is the first since Marx’s single-centre study [10] published over three decades ago. The trial will provide data on the incidence of ORN after dental extraction and implant placement as well as the effect of HBO on retention of implants. Importantly, HOPON will provide translational resource for future studies of the genomic determinants of ORN in a group of patients treated to a defined protocol.

### Trial status

Feasibility and interim analysis complete. Patient recruitment and data collection is ongoing to Protocol 9, dated 11 January 2017.

## Additional file

Additional file 1: SPIRIT 2013 Checklist: recommended items to address in a clinical trial protocol and related documents. (DOC 121 kb)

## Abbreviations

ATA: Atmospheres
GCLP: Good Clinical Laboratory Practice
HBO: Hyperbaric oxygen
HOPON: Hyperbaric Oxygen for the Prevention of Osteoradionecrosis
IMRT: Intensity-modulated radiotherapy
ISDMC: Independent Safety Data Monitoring Committee
ITT: Intention-to-treat
MBS: Minor bone spicules
MHRA: Medicines and Healthcare Products Regulatory Authority
NICE: National Institute for Health and Care Excellence
ORN: Osteoradionecrosis
PI: (Site) principal investigator
PpO_2_: Partial pressure of oxygen
RCT: Randomised controlled trial
SPIRIT: Standard Protocol Items Recommendations for Interventional Trials
TSC: Trial Steering Committee
